# Supramaximal-Exercise Training Improves Heart Rate Variability in Association With Reduced Catecholamine in Obese Adults

**DOI:** 10.3389/fphys.2021.654695

**Published:** 2021-03-30

**Authors:** Georges Jabbour, Horia D. Iancu

**Affiliations:** ^1^Physical Education Department, College of Education, Qatar University, Doha, Qatar; ^2^School of Kinesiology and Leisure, University of Moncton, Moncton, NB, Canada

**Keywords:** low-frequency, high-frequency, waist-to-hip ratio, noradrenaline, maximal oxygen consumption, ventilatory thresholds 1

## Abstract

This study investigates the effect of 6 weeks of supramaximal exercise training (SET) on heart rate variability (HRV) and associated factors in sedentary obese (OB) and normal-weight (NW) adults. In this study, 19 OB [22.9 (8.4) years; body mass index (BMI) 33.4 (1.4) kg/m^2^] and 18 NW [23.2 (4.4) years; BMI 23.3 (1.2) kg/m^2^] adults completed a 6-week SET intervention. Anthropometric and aerobic indicators as well the homeostasis model assessment index for insulin resistance index (HOMA-IR) were assessed at baseline and after SET. The low- and high-frequency [(LF (0.03–0.15 Hz) in ms^2^ and HF (0.15–0.4 Hz) in ms^2^)] analysis of HRV as well as adrenaline (A in nmol/l) and noradrenaline (NA in nmol/l) responses were assessed at resting condition and during ventilatory threshold 1 (VT1) of a graded maximal test at baseline and after SET. At baseline, resting HF, LF and the LF/HF ratio were different among groups (*P* < 0.01, respectively) and were significantly associated with waist-to-hip ratio (β = −0.26; *p* = 0.01, β = −0.12; *p* = 0.01 and, β = 0.21; *p* = 0.01). During exertion at VT1, only LF/HF ratio was associated with NA responses (β = 0.23; *p* = 0.01). After SET, the frequency domain marker improved significantly for both groups in comparison to baseline. These improvements are manifested by LF and HF increases and LF/HF ratio decreases in the rest condition (*p* < 0.01, respectively) and during exertion at VT1 (*p* < 0.01, respectively). The improvement in LH and HF were associated with VO_2_max increases (β = 0.22 *p* = 0.01 and β = 0.33; *p* = 0.01). The decreases observed for the LF/HF ratio are mainly associated to NA decreases observed at rest (β = 0.31; *p* = 0.001) and at VT1 (β = 0.38; *p* = 0.001). Obese adults have altered HRV, and 6 weeks of SET improves HRV variables at rest and during VT1 exertion. While LF and HF improvement were associated with VO2max increases, the LF/HF ratio was mainly associated with noradrenaline decreases observed at rest and at VT1.

## Introduction

Obesity has been shown to be a major public health problem with serious psychological and medical consequences due to comorbidities such as metabolic disorder (Hubert et al., 1983), cardiovascular diseases (Jensen et al., 2014), “resistance” or loss of sensitivity (loss of response per unit of hormone) for several hormones (McMurray and Hackney, 2005), and a dysregulation of cardiac autonomic function (ANS) (Lindmark et al., 2005). This last problem might be an important mediator in the development of cardiovascular disease (CVD) risks among the obese population (Sheema and Malipatil, 2015).

The assessment of heart rate variability (HRV) has been accepted as a non-invasive and useful tool in estimating autonomic function [Task Force of the European Society of Cardiology and the North American Society of Pacing and Electrophysiology [TFESCNASPE], 1996]. Indeed, the high frequency (HF) is associated solely with parasympathetic nervous system (PNS) activity, whereas low frequency (LF) is thought to reflect solely sympathetic activity (SNS) (Malliani et al., 1991) or to be an index of both vagal and sympathetic activity (Pomeranz et al., 1985; Cook et al., 1991). It is well known that with obesity, both the SNS and PNS are altered with a significant change in vagosympathetic activity (Petretta et al., 1995; Gutin et al., 2005; Tian et al., 2015). In comparison to normal weight individuals, obese subjects showed lower HRV with significant increases in SNS activity and reduced modulation of PNS (Lindmark et al., 2005; Sheema and Malipatil, 2015). This impairment reflects poor adaptation of the autonomic nervous system in response to physiological stress, such as physical exertion (Goldstein et al., 2011; Massote et al., 2019). However, exercise interventions improve HRV (Phoemsapthawee et al., 2019) mainly by improving PNS activity with a concomitant decrease in SNS activity (Tian et al., 2015). Studies associated HRV improvement to body composition and to fitness improvement (Gutin et al., 2005; Tian et al., 2015), to insulin resistance (Lindmark et al., 2005), leptin (Quilliot et al., 2008), and inflammation (Akinci et al., 2008) decreases.

On the other hand, the ANS plays an important role in metabolism control, and any impairment in its activity may contribute to the pathogenesis of obesity (Peterson et al., 1988**;** Bray, 1991). In line with the above, several studies conducted in obese individuals reported a significant impairment in sympathetic nervous system (SNS) activity evaluated by higher adrenaline (A) and noradrenaline (NA) concentrations at rest (Troisi et al., 1991; Jones et al., 1997), with a reduced metabolic response (Seals and Bell, 2004). Actually, SNS and PNS, branches of the nervous system, act by the intermediary of their secreted hormones (e.g., catecholamine), and previous investigations using HRV analysis and measurements of plasma catecholamines (Gustafson and Kalkhoff, 1982; Jung et al., 1982) showed impaired sympathetic responsiveness during cold exposure, consumption of spicy food containing capsaicin and glucose ingestion in obese individuals. Breuer et al. (1993) attempted to assess whether HRV is associated with catecholamine concentrations during exercise. For these authors, an additional reduction in LF during exercise at 150 beats per minute of the heart rate reflect a negative feedback of circulating catecholamines on the sympathetic control of heart rate. Similarly, Kienzle et al. (1992) showed a significant relation between HRV and plasma noradrenaline concentration in patients with congestive heart failure. Despite this promising research track, no previous work has attempted to elucidate the link between HRV and catecholamine responses in obese individuals in response to an exercise intervention.

Because obesity is associated with an impairment of HRV and catecholamine responses, the present work investigates the potential link between HRV and catecholamine responses before and after an exercise intervention. Specifically, we studied whether 6 weeks of supramaximal exercise training (SET) could improve cardiac autonomic modulation and whether this improvement was associated to circulating catecholamine in parallel to the other conventional variables previously studied (e.g., anthropometric parameters, metabolic adaptations and fitness level). We hypothesized that a reduction of HRV might be observed in obese adults in comparison to normal-weight ones and SET may improve HRV in obese adults. This improvement may be associated with catecholamines decreases.

## Materials and Methods

### Participants and Study Design

**FIGURE 1:**
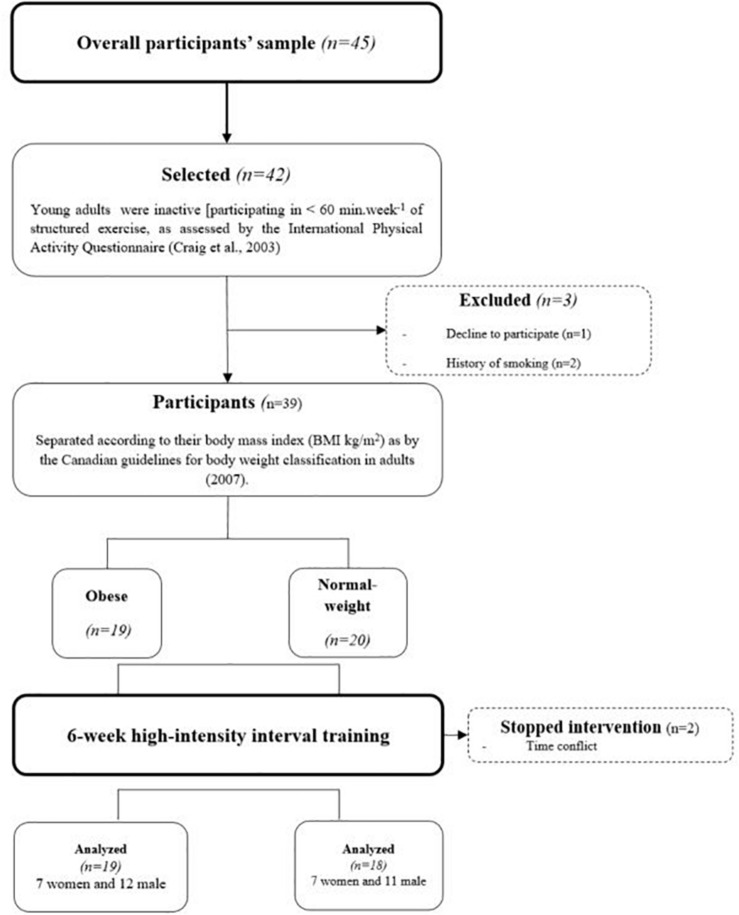
Flowchart of participants’ recruitment.

The study followed the STROBE (STrengthening the Reporting of OBservational studies in Epidemiology) checklist and is listed on the ISRCTN registry with study ID [ISRCTN17120788 (01/05/2020)]. Thirty-seven young adults [18 normal-weight NW (seven women and 11 men): 23.2 (4.4) years and 19 obese (seven women and 12 men): 22.9 (8.4) years were recruited from the Moncton campus of the Université de Moncton (Figure 1). Announcements were posted throughout the University campus, and we invited students who met the inclusion criteria to voluntarily participate in the project. The study protocol was approved by the University’s Human Research Ethics Committee (UHRC) mandated by the Faculty of Higher Studies (under the number: 1,415–071) and all procedures were followed in accordance with the Helsinki Declaration of 1975, as revised in 2008. Informed consent was obtained from all subjects prior to being included in the study.

The inclusion criteria for participation were as follows: participants had to be inactive [participating in <60 min week-1 of structured exercise, as assessed by the International Physical Activity Questionnaire (Craig et al., 2003), and none of them took part in any systematic exercise training at the time of study enrollment or during the 6 months that preceded the experiment. Moreover, they had no history of cardiovascular or other chronic disease, no history of drug consumption before the study, and no history of smoking. Consequently, out of overall participants’ sample (*n* = 45), 42 were initially selected, but three were excluded for history of smoking (*n* = 1) or declining to participate (*n* = 2). Before entering this protocol, each participant was thoroughly familiarized with all testing equipment and procedures. The protocol then began with two sessions of preliminary testing to determine baseline levels of key variables (baseline testing). The testing was conducted in the laboratory rooms of the Université de Moncton campus on two different days (day one—D1 and day two—D2) after an overnight fast and took place in the morning of each day (∼8 h 30). The two testing days were separated by a minimum of 48 h, and all subjects were asked to avoid physical activity for 48 h prior to each session. The participants recorded a 48-h food diary before intervention at the baseline and during the final days of testing (post-intervention). During the 6 weeks of intervention, only two NW participants interrupted the training sessions because of a time conflict related to university studies, but no other difficulties were encountered.

### Anthropometric Measurements

The assessment of body mass, body fat percentage, fat-free mass (FFM) and fat mass was done using a bioimpedance machine (Vacumed, Bodystat1500, Isle of Man, British Isles). With a wall-mountable height rod (HR-200 Tanita, IL, United States), height was determined to the nearest 0.5 cm. BMI was calculated as the ratio of mass (kg) to height squared (m^2^). Waist conference (WC) and hip circumference (HC) were measured twice to the nearest 0.5 cm with a flexible tape. WC was measured at the level of the natural waist (the narrowest part of the torso) or one finger width below the umbilicus. HC was measured at the maximum circumference of the buttocks posteriorly and the symphysis pubis anteriorly in a horizontal plane [World Health Organization [WHO], 2008]. Waist-to-hip ratio was calculated using the following formula: Waist-to-hip ratio = WC (cm)/HC (cm).

### Calculation of Metabolic and Physiological Variables

At baseline on D1, after a 12-h overnight fast, each participant reclined in a supine position for 10 min before venous blood samples from an antecubital vein were drawn at rest and collected in a vacutainer tube containing ethylene diamine tetra acetic acid (EDTA). During this period, the heart rate were continuously measured using an electrocardiogram machine (CASE 16 exercise testing system, Marquette, WI, United States). Subsequently, participants performed a maximal test on an upright cycle ergometer (Monark Ergomedic 839E electronic test cycle, Sweden) with continuous measurement of pulmonary gas exchange using a breath-by-breath automated metabolic system (Ergocard MEDI-SOFT, Sorinnes, Belgium) to determine their maximal oxygen consumption (VO2max) according to criteria established by Spiro (1979).

The test began with an initial power of 25 W and was progressively increased by 25 W every 5 min until exhaustion. During this maximal test, the heart rate were continuously measured using an electrocardiogram machine (CASE 16 exercise testing system, Marquette, WI, United States). For the purpose of the present work, ventilatory threshold 1 (VT1) was determined using established criteria as per Wasserman et al. (1999).

Briefly, VT1 corresponds to the break point in the plot of VCO2 as a function of VO2. At that point, VE/VO2 increases without an increase in VE/VCO2. Three experienced investigators determined VT1 independently. To determine the VO2 and VE at VT1, the average of the last 20 s of the corresponding level was used. The energy consumption, (E) in watts, was calculated as follows: E = (4.94 RER + 16.04) × VO2net/60.37 Net VO2 (VO2net, L/min) was calculated by subtracting the resting value from the gross value at each intensity stage. The E was assessed at rest and at VT1 for both groups and was realized at baseline and at post-intervention.

On D2, after 10 min of warm-up, subjects performed a Force-Velocity test. The Force-Velocity test was performed on a cycle ergometer using a technique adapted from the study of Vandewalle et al. (1988). This test consists of a succession of supramaximal bouts of approximately 6 s with the exercise loads increasing by 1 kg after each bout until inability to pursue the test. A passive recovery (5 min) was allowed between successive bouts. The velocity for each bout was recorded every 2 s. Only the highest velocity was recorded for each load using an electronic counter (MEV 2000). Power output was calculated by multiplying load and speed, and a power curve was then compiled for each bout. The optimal load corresponding to maximal power (Pmax) was used for the training protocol that followed.

Once participants completed the preliminary testing, a total of 18 training sessions was prescribed for the training group (three sessions per week for 6 weeks). To facilitate the task, half of participants underwent the training session on Monday, Wednesday, and Friday and the other half on Tuesday, Thursday, and Saturday. Each of the prescribed sessions began with a 5-min warm-up of continuous cycling at moderate intensity corresponding to 40–50% of their HRmax and was followed by six repetitions of supramaximal exercise intervals with 2 min of passive recovery between each repetition. This relatively new form of very short supramaximal cycling exercise (6 s), has been shown to be very appealing and well tolerable for our previous experimental participants (Jabbour et al., 2016). Throughout the intervention, the subjects were asked to refrain from consuming alcohol and encouraged to continue their normal diet and maintain their typical sedentary behavior.

Plasma epinephrine and norepinephrine concentrations were measured using high-performance liquid chromatography (HPLC; Chromsystems; Thermo Finnigan, France) following the method of Koubi et al. (1991). The method is fully described in our previous works (Jabbour and Majed, 2019). Plasma glucose concentrations were determined using commercially available kits (all ABX Pentra, Montpellier, France). A single analyzer run was used for each subject, and each sample was analyzed twice. The intra-assay coefficient of variation (CV) was 1.65%. Plasma insulin concentrations were measured in the centralized laboratory by a radioimmunoassay procedure (Phadebas Insulin Kit; Pharmacia Diagnostics AB, Piscataway, NJ, United States). The insulin resistance was estimated by the homeostasis model assessment (HOMA-IR) index as [fasting insulin (μU/ml) × fasting glucose (mmol/l)]/22.5.35 Plasma hormones and metabolites values were corrected for plasma volume changes using the equation of Van Beaumont (1972).

For the purpose of the present work, these measurements were performed at baseline, at rest condition, and at VT1 for both groups and were repeated post-intervention.

### Spectral Analysis of Heart Rate Variability

Spectral analyses of HRV were evaluated at rest and during exercise at intensity corresponding to ventilator threshold 1 in obese and normal weight adults at D1. Resting measures were collected for 5 min while participants were in a quit rest (supine position) before the commencement of maximal test. Participants were instructed to maintain their breathing rate at ∼12 breaths per minute during the recordings and were called continually to breathe comfortably without hyperventilating or substantially changing the depth of their breathing.

Using an electrocardiogram (CASE 16 exercise testing system, Marquette, WI, United States) the R waves of electrocardiograms were identified beat-by-beat, and the pulse interval was computed as the interval between two consecutive beats using software (Kubios HRV Standart for Windows 3.0.2). The frequency-domain analyses were evaluated in both groups at rest, at VT1, and at baseline, and repeated post-intervention. Fast Fourier transformation and Hanning windows (512) with 50% overlap obtained the power spectral density. The spectral power for low frequency (LF = 0.04–0.15 Hz) and high frequency (HF = 0.15–0.4 Hz) bands were evaluated. To assess the sympathovagal balance to the heart, the LF/HF ratio of cardiac interval variability was calculated.

#### Statistical Analysis

Data are presented as the mean and standard deviation (SD). On the basis of a power analysis (desired power = 0.85 and an alpha error = 0.05) of variables of interest, we determined that a sample size of *n* = 8 per group would be sufficient to detect differences between groups for values obtained pre- and postintervention [(population *r* = 0.59, tested against a constant of 0.00, sample size (8), and alpha (0.059, 2-tailed), power is 0.813)].

Before the analysis, all datasets were tested for normality using the Kolmogorov–Smirnov test. ANOVAs with 2 × 2 repeated measures [Intervention (pre- and post-SET intervention) × Group (NW and OB)] were performed for all dependent variables at rest and at VT1. When a significant main effect was found, a *post hoc* analysis using Bonferroni’s test was performed for pairwise comparisons. Cohen’s d effect sizes (ES) were also calculated and interpreted as small (*d* = 0.2), medium (*d* = 0.5), or large (*d* = 0.8).

We used multiple linear regression to model the mean outcomes for each exposure of interest. For both linear and logistic regressions, the independent variables considered in the regression models were frequency-domain analyses (i.e., Rest and VT1).

## Results

The ANOVA results on anthropometric variables are presented in Table 1. At the baseline test, an initial significant difference in body mass, BMI, FM and waist-to-hip ratio values was detected between the NW and OB groups and maintained at the post-intervention test. For the OB subjects body mass and the BMI significantly decreased in comparison to baseline (*p* < 0.01, respectively) (Table 1). No significant changes were induced by the SET program in NW group. At the pre-intervention phase, the OB group had significantly higher values for fasting blood sugar and HOMA-IR at rest, while no other differences were noted. The SET intervention significantly improved the SBP, fasting blood sugar and HOMA-IR values for the OB group at rest, while no intervention-related changes were found for other physiological and fitness variables. Moreover, the initial between-group differences in fasting blood sugar and HOMA-IR rest were no longer significant at post-intervention (Table 1).

**TABLE 1:** Age, anthropometric and fitness data at pre and after 6-week supramaximal exercise training.

	**Preintervention (baseline)**		**Postintervention**		**Group × Time effects**		**95% CI**	**Effect Size**
	**Normal-weight (*n* = 18) (*w* = 7, *m* = 11)**	**Obese (*n* = 19) (*w* = 7, *m* = 12)**	**Normal-weight (*n* = 18) (*w* = 7, *m* = 12)**	**Obese (*n* = 19) (*w* = 7, *m* = 12)**	***F***	***P***		**Cohen’s *d***
Age (year)	23.2 (4.4)	22.9 (8.6)	–	–	1.8	0.21	–	–
Height (m)	171.6 (6.4)	170.9 (8.3)	171.6 (6.4)	170.9 (8.3)	1.4	0.38	–	0.91
Body mass (kg)	68.2 (4.3)	99.5 (11.1)^a^	67.29 (1.3)	92.3 (4.1)^a b^	21.6	<0.01	2.1(0.4 to 4.1)	0.41
BMI (kg/m^2^)	23.3 (1.2)	33.4 (1.4)^a^	23.01 (2.6)	31.6 (1.5)^a b^	12.7	<0.01	4.1(0.6 to 3.9)	0.32
FM (%)	17.9 (3.2)	42.1 (4.2)^a^	17.3 (2.8)	42.9 (0.9)^a^	20.1	<0.01	3.9(0.9 to 2.9)	0.23
Waist-to-hip ratio	0.78 (0.01)	0.92 (0.08)^a^	0.79 (0.05)	0.93 (0.03)^a^	35.7	<0.01	0.22 (−0.19 to 0.46)	0.54
VO_2max_ (ml/min/kg)	23.5 (5.6)	24.1 (6.0)	25.7 (1.9)^b^	26.2 (2.3)^b^	14.3	<0.05	8.3(−0.4 to 16.4)	0.52
HR max *(beats/min)*	196.9 (4.1)	195.3 (4.1)	193.8 (3.1)	197.1 (2.9)	0.5	0.61	11.6 (−21.3 to 31.3)	0.81
Fasting blood sugar (mmol/l)	4.19 (0.19)	4.91 (0.09)^a^	4.09(1.09)^b^	4.13 (0.31)^b^	19.3	<0.05	0.29 (−0.14 to 0.36)	0.56
HOMA-IR	1.79 (0.8)	4.51 (1.1)^a^	1.65 (0.7)	2.82 (1.2)^b^	10.9	<0.01	0.19 (−0.11 to 0.12)	0.88

*Values are mean ± SE (standard error). w, woman; m, men; CI, confidence of interval; BMI, body mass index; FM, fat mass; VO_2max_, maximal oxygen consumption; HRmax, maximal heart rate; HOMA-IR, homeostasis model assessment index for insulin resistance.*

*Significant difference between groups (^*a*^*p* < 0.01), Significant difference from baseline values (^*b*^*p* < 0.01).*

At the baseline test, the OB subjects presented lower values of HF and LF (*p* < 0.01, respectively) than the NW subjects, while LF/HF was higher in the OB group (*p* < 0.01) (Table 2). Moreover, resting values of oxygen and energy consumption as well as for the heart rate, adrenaline, and noradrenaline were significantly higher for the OB group than for the NW group (*p* < 0.01, respectively). After SET, HF and LF increased for both groups compared to baseline (*p* < 0.01, respectively). However, the LF/HF ratio decreased significantly (*p* < 0.01, respectively). Energy consumption, adrenaline, noradrenaline and SBP also decreased post-SET for both groups (*p* < 0.01, respectively) (Table 2). Obese individuals showed higher values of VO2, E and NA than the NW participants (Table 2). The OB group also showed significant decreases in oxygen consumption and HR (*p* < 0.01, respectively).

**TABLE 2:** Frequency domain marker, fitness and hormonal parameters measured at baseline and after 6-week supramaximal exercise training.

	**Preintervention (baseline)**		**Postintervention**		**Group × Time effects**		**95% CI**	**Effect size**
	**Normal-weight (*n* = 18) (w = 7, m = 11)**	**Obese (*n* = 19) (w = 7, m = 12)**	**Normal-weight (*n* = 18) (w = 7, m = 11)**	**Obese (*n* = 19) (w = 7, m = 12)**	***F***	***P***		**Cohen’s *d***
**At rest condition**								
LF (ms^2^)	1,320 (277)	999 (677)^a^	1,760 (830)^b^	1,880 (698)^b^	11.6	<0.01	21.9 (−60.5 to 236.9)	0.61
HF (ms^2^)	1,730 (892)	1,371 (791)^a^	2,111 (881)^b^	2,480 (448)^b^	21.7	<0.01	71 (−189 to 522)	0.56
LF/HF ratio	0.85 (0.7)	1.93 (1.15)^a^	0.73 (0.2)^b^	0.83 (0.91)^a b^	6.6	<0.01	2.4(−2.1 to −1.3)	0.44
VO_2_ (ml/min)	526 (12)	638 (12)^a^	499 (28)	615 (11)^a^	19.7	<0.01	3.6(−0.9 to 12.8)	0.66
E (Watt)	76 (11)	205 (18)^a^	66 (9)^b^	150 (14)^a b^	13.9	<0.01	2.1(−1.9 to −1.8)	0.46
HR (beat/minute)	66 (8)	81 (8)^a^	65 (11)	69 (15)^b^	18.1	<0.01	0.7(−1.2 to −0.3)	0.36
Adrenaline (nmol/l)	1.5 (0.01)	2.1 (0.09)^a^	1.1 (0.1)^b^	1.3 (0.03)^b^	10.8	<0.01	2.1(0.2 to 1.9)	0.51
Noradrenaline (nmol/l)	6.2 (2.1)	11.8 (4.1)^a^	5.8 (1.3)^b^	7.8 (2.8)^a b^	21.1	<0.01	3.4(−2.2 to −1.1)	0.59
HR (beats/minute)	76 (6)	96 (8)^a^	74 (3)	79 (9)^b^	14.5	<0.01	9.6(−11.1 to 21.1)	0.65
SBP (mmHg)	125 (1.5)	121 (3.1)	112 (2.1)^b^	113 (2.2)^b^	17.3	<0.01	2.2(−1.9 to −0.8)	0.32
DBP (mmHg)	79 (5)	76 (6)	77 (1)	76 (3)	1.5	0.61	1.8 (−3.4 to −0.4)	0.39
**Intensity corresponding to ventilator threshold 1 (VT1)**								
LF (ms^2^)	970 (232)^c^	880 (227)^c^	1,266 (812)^bc^	1,328 (444)^bc^	*31.2*	<0.01	33.7 (−70.1 to 361.2)	0.31
HF (ms^2^)	1,201 (698)^c^	1,088 (851)^c^	1,602 (901)^bc^	1,720 (779)^b c^	*28.5*	<0.01	59 (−201 to 499)	0.46
LF/HF ratio	1.05 (0.7)^c^	3.49 (1.68)^a c^	0.83 (0.9)^b^	0.93 (0.3)^b^	*17.7*	<0.01	1.9(−2.3 to −2.6)	0.55
VO_2_ (ml/min)	962 (44)^c^	1,668 (52)^a c^	424 (21)^b c^	811 (43)^a b c^	*16.5*	<0.01	2.9(−1.1 to 7.7)	0.66
E (Watt)	589 (21)^c^	835 (14)^ac^	369 (33)^b c^	669 (19)^a b c^	*19.6*	<0.01	2.6(−1.7 to −2.1)	0.56
Adrenaline (nmol/l)	1.71 (0.2)^c^	1.91(0.3)^c^	1.45 (0.3)^b c^	1.58 (0.5)^b c^	*9.9*	0.68	1.7 (−2.2 to −0.8)	0.64
Noradrenaline (nmol/l)	7.6 (1.1)^c^	14.9 (3.1)^a c^	6.6 (1.1)^b c^	9.1 (1.1)^a b c^	*23.3*	<0.01	4.1(0.6 to 3.3)	0.78
HR (beat/minute)	117 (12)^c^	139 (13)^a c^	109 (2)^b c^	118 (3)^b c^	*17.3*	<0.01	1.9(−1.7 to −2.1)	0.14
SBP(mmHg)	154 (9)^c^	170 (14)^a c^	155 (10)^c^	158 (5)^b c^	*19.1*	<0.01	6.2(−10.2 to 11.4)	0.09
DBP(mmHg)	85(3)^c^	88 (3)^c^	79 (7)^c^	78 (9)^c^	*2.9*	0.78	1.6 (−1.7 to −1.1)	0.07

*Values are mean ± SE (standard error). w, woman; m, men; CI, confidence of interval; HF, high-frequency power; LF, low-frequency power; LF/HF ratio, low-frequency to high-frequency ratio; VO_2_, oxygen consumption; E, energy consumption; HR, heart rate; SBP, Systolic blood pressure; DBP, diastolic blood pressure.*

*Significant difference between groups (^*a*^*p* < 0.01), significant difference from baseline values (^*b*^*p* < 0.01), and significant difference with rest (^*c*^*p* < 0.01).*

For the VT1 exercise condition, there were significant increases in HF and LF and decreases in LF/HF ratios compared to rest for both groups (Table 2). The OB group had lower HF, LF and higher LF/HF ratio than the NW group (*p* < 0.01, respectively). Moreover, oxygen and energy consumption as well as heart rate, noradrenaline and SBP were significantly higher in the OB group than in the NW group (*p* < 0.01, respectively). After SET, HF increased for both groups (*p* < 0.01, respectively) while LF and the LF/HF ratio decreased significantly compared to baseline (*p* < 0.01, respectively). Moreover, plasma adrenaline, noradrenaline, and HR decreased significantly for both groups post-SET (*p* < 0.01, respectively). For oxygen consumption, energy consumption and noradrenaline concentration, the OB group showed significantly higher values post-intervention compared to the NW group (Table 2).

Finally, the VO2max did not differ among group at baseline condition. After SET, VO2max increased for both groups compared to baseline and were similar between OB and NW subjects (Table 2).

At baseline (Table 3), our regression models reported a significant negative correlation between the rest value of HF and of LF and a significant positive association between the LF/HF ratio with Waist-to-hip ratio. At the VT1 condition, only the LF/HF ratio correlate positively with noradrenaline concentration. At post-intervention, resting HF and LF correlated with VO2max. Moreover, HF and LF determined at VT1correlated significantly with VO2max. LF/HF ratio correlated with noradrenaline, fasting blood sugar and HOMA-IR levels. At the VT1 condition, there were significant correlations between LF/HF decreases and noradrenaline concentration (Table 3). Finally, at VT1, LF correlated negatively HR.

**TABLE 3:** Standardized regression summary between frequency domain markers and anthropometric, fitness and biochemical variables in both groups.

	**Baseline**			**Post-intervention**		
	**HF**	**LF**	**LF/HF ratio**	**HF**	**LF**	**LF/HF ratio**
Body mass *(kg)*	*R*^2^ adj. = 0.04; *p* = 0.51	*R*^2^ adj. = 0.09; *p* = 0.43	*R*^2^ adj. = 0.03; *p* = 0.36	*R*^2^ adj. = 0.05; *p* = 0.67	*R*^2^ adj. = 0.08; *p* = 0.48	*R*^2^ adj. = 0.06; *p* = 0.48
BMI *(kg/m^2^)*	*R*^2^ adj. = 0.06; *p* = 0.62	*R*^2^ adj. = 0.05; *p* = 0.54	*R*^2^ adj. = 0.03; *p* = 0.49	*R*^2^ adj. = 0.05; *p* = 0.48	R^2^ adj. = 0.06; *p* = 0.38	*R*^2^ adj. = 0.07; *p* = 0.56
FM *(%)*	*R*^2^ adj. = 0.06; *p* = 0.64	*R*^2^ adj. = 0.04; *p* = 0.43	*R*^2^ adj. = 0.04; *p* = 0.39	*R*^2^ adj. = 0.05; *p* = 0.43	*R*^2^ adj. = 0.07; *p* = 0.64	*R*^2^ adj. = 0.09; *p* = 0.62
Waist-to-hip ratio	*R*^2^ adj. = −0.36; *p* = 0.01*	*R*^2^ adj. = −0.22; *p* = 0.01*	*R*^2^ adj. = 0.23; *p* = 0.01*	*R*^2^adj. = 0.07; *p* = 0.52	*R*^2^ adj. = 0.04; *p* = 0.33	*R*^2^ adj. = 0.06; *p* = 0.52
**At rest condition**						
VO_2_ *(ml/min)*	*R*^2^ adj. = 0.03; *p* = 0.58	*R*^2^ adj. = 0.04; *p* = 0.48	*R*^2^ adj. = 0.06; *p* = 0.42	*R*^2^ adj. = 0.04; *p* = 0.36	*R*^2^ adj. = 0.03; *p* = 0.48	*R*^2^ adj. = 0.08; *p* = 0.42
E *(Watt)*	*R*^2^ adj. = 0.07; *p* = 0.64	*R*^2^ adj. = 0.06; *p* = 0.45	*R*^2^ adj. = 0.06; *p* = 0.63	*R*^2^ adj. = 0.08; *p* = 0.38	*R*^2^ adj. = 0.07; *p* = 0.51	*R*^2^ adj. = 0.09; *p* = 0.32
Waist-to-hip ratio	*R*^2^ adj. = −0.36; *p* = 0.01*	*R*^2^ adj. = −0.22; *p* = 0.01*	*R*^2^ adj. = 0.23; *p* = 0.01*	*R*^2^adj. = 0.07; *p* = 0.52	*R*^2^ adj. = 0.04; *p* = 0.33	*R*^2^ adj. = 0.06; *p* = 0.52
Adrenaline *(nmol/l)*	*R*^2^ adj. = 0.06; *p* = 0.32	*R*^2^ adj. = 0.06; *p* = 0.61	*R*^2^ adj. = 0.05; *p* = 0.52	*R*^2^ adj. = 0.04; *p* = 0.48	*R*^2^ adj. = 0.06; *p* = 0.42	*R*^2^ adj. = 0.08; *p* = 0.32
Noradrenaline *(nmol/l)*	*R*^2^ adj. = 0.08; *p* = 0.54	*R*^2^ adj. = 0.08; *p* = 0.14	*R*^2^ adj. = 0.04; *p* = 0.19	*R*^2^ adj. = **−**0.04; *p* = 0.15	*R*^2^ adj. = **−**0.08; *p* = 0.11	*R*^2^ adj. = 0.32; *p* = 0.01*
Fasting blood sugar *(mmol/l)*	*R*^2^ adj. = 0.06; *p* = 0.31	*R*^2^ adj. = 0.08; *p* = 0.41	*R*^2^ adj. = 0.06; *p* = 0.42	*R*^2^ adj. = 0.06; *p* = 0.23	*R*^2^ adj. = 0.03; *p* = 0.21	*R*^2^ adj. = 0.31; *p* = 0.01*
HOMA-IR	*R*^2^ adj. = 0.06; *p* = 0.05	*R*^2^ adj. = 0.06; *p* = 0.21	*R*^2^ adj. = 0.04; *p* = 0.42	*R*^2^ adj. = 0.08; *p* = 0.55	*R*^2^ adj. = 0.04; *p* = 0.22	*R*^2^ adj. = 0.19; *p* = 0.05*
**Intensity corresponding to ventilator threshold 1 (VT1)**						
Body mass *(kg)*	*R*^2^ adj. = 0.07; *p* = 0.21	*R*^2^ adj. = 0.07; *p* = 0.33	*R*^2^ adj. = 0.04; *p* = 0.31	*R*^2^ adj. = 0.04; *p* = 0.47	*R*^2^ adj. = 0.06; *p* = 0.34	*R*^2^ adj. = 0.07; *p* = 0.55
BMI *(kg/m^2^)*	*R*^2^ adj. = 0.05; *p* = 0.42	*R*^2^ adj. = 0.07; *p* = 0.44	*R*^2^ adj. = 0.06; *p* = 0.39	*R*^2^ adj. = 0.06; *p* = 0.36	*R*^2^ adj. = 0.07; *p* = 0.22	*R*^2^ adj. = 0.06; *p* = 0.66
FM *(%)*	*R*^2^ adj. = 0.06; *p* = 0.64	*R*^2^ adj. = 0.08; *p* = 0.53	*R*^2^ adj. = 0.06; *p* = 0.29	*R*^2^ adj. = 0.08; *p* = 0.71	*R*^2^ adj. = 0.07; *p* = 0.61	*R*^2^ adj. = 0.07; *p* = 0.59
Waist-to-hip ratio	*R*^2^ adj. = 0.05; *p* = 0.34	*R*^2^ adj. = 0.06; *p* = 0.55	*R*^2^ adj. = 0.04; *p* = 0.23	*R*^2^ adj. = 0.06; *p* = 0.66	*R*^2^ adj. = 0.06; *p* = 0.54	*R*^2^ adj. = 0.06; *p* = 0.44
VO_2_ *(ml/min)*	*R*^2^ adj. = 0.04; *p* = 0.35	*R*^2^ adj. = 0.06; *p* = 0.43	*R*^2^ adj. = 0.07; *p* = 0.63	*R*^2^ adj. = 0.06; *p* = 0.62	*R*^2^ adj. = 0.05; *p* = 0.31	*R*^2^ adj. = 0.06; *p* = 0.49
E *(Watt)*	*R*^2^ adj. = 0.05; *p* = 0.51	*R*^2^ adj. = 0.07; *p* = 0.43	*R*^2^ adj. = 0.08; *p* = 0.59	*R*^2^ adj. = 0.06; *p* = 0.41	*R*^2^ adj. = 0.06; *p* = 0.31	*R*^2^ adj. = 0.06; *p* = 0.22
Adrenaline *(nmol/l)*	*R*^2^ adj. = 0.05; *p* = 0.33	*R*^2^ adj. = 0.08; *p* = 0.34	*R*^2^ adj. = 0.08; *p* = 0.35	*R*^2^ adj. = 0.07; *p* = 0.77	*R*^2^ adj. = 0.08; *p* = 0.66	*R*^2^ adj. = 0.06; *p* = 0.43
Noradrenaline *(nmol/l)*	*R*^2^ adj. = 0.06; *p* = 0.29	*R*^2^ adj. = 0.05; *p* = 0.21	*R*^2^ adj. = 0.31; *p* = 0.01*	*R*^2^ adj. = 0.06; *p* = 0.15	*R*^2^ adj. = 0.03; *p* = 0.31	*R*^2^ adj. = 0.39; *p* = 0.001*

HF, high-frequency power; LF, low-frequency power; LF/HF ratio, low-frequency to high-frequency ratio; BMI, body mass index; FM, Fat Mass; VO_2_, oxygen consumption; VO_2max_, maximal oxygen consumption; E, energy consumption; HOMA-IR, homeostasis model assessment index for insulin resistance.

*denotes significant relationship. Bold values more visible for statistical significance.

## Discussion

To the best of our knowledge, the current study is the first that examined the association between HRV indices, mainly HF, LF and the LF/HF ratio, with catecholamine levels and other conventional factors among obese and normal weight adults. The variabilities in frequency domain indices as well as in catecholamines have been evaluated at rest and during exertion at VT1 of a graded maximal exercise before and after 6 weeks of SET. Despite that studying HRV as an accurate tool to assess sympathetic modulation still controversial (Goldstein et al., 2011), the main finding of the present study was that obese individuals presented significant differences in frequency domain at rest and during VT1 intensity compared to normal weight individuals, indicating an existing impairment in autonomic nervous system modulation. After 6 weeks of SET, there was a significant improvement in HRV indicators associated with VO2max improvement for HF and LF and with noradrenaline decreases and HOMA-IR improvement for LF/HF ratio.

At rest, our study confirmed that HF and LF indices in milliseconds squared, were lower in our obese group than in the normal weight group. Moreover, the LF/HF ratio were increased in obese subjects in comparison to normal weight controls. Our result agrees with previous studies showing impairment in autonomic responses among obese individuals (Lindmark et al., 2005; Yadav et al., 2017). The impaired autonomic modulation observed among the obese population is mainly associated to the increased waist-to-hip ratio (Yadav et al., 2017), a reliable indicator of abdominal obesity. Thus, our findings showed that waist-to-hip ratio associated negatively with HF and LF and positively with LF/HF ratio in the obese group, a result that is also supported by Yadav et al. (2017). In fact, an increase in abdominal fat, responsible for secreting certain cytokines and chemicals, alters autonomic balance (Prasad et al., 2011) without excluding other potential factors (eg., insulin resistance, fasting glucose level) reported among diabetes or heart diseases patients (Singh et al., 2000). In the present work, no association was found between insulin resistance estimated by HOMA-IR and blood glucose level.

However, the increased concentration of noradrenaline associates significantly with the LF/HF ratio. Indeed, an increase in noradrenaline concentration and a decrease in adreno-receptor responsiveness are common in obese individuals with or at risk of a metabolic disorder. Within this context, many authors suggested that in such abnormal cases, the parasympathetic tone withdrawal and/or sympathetic activity increased (Ramaekers et al., 1998; Fraley et al., 2005). The association between catecholamine with HRV has been rarely studied and mostly performed under a pharmacological setting (e.g., troponin vs. noradrenaline infusion) or in response to a stimulus (e.g., cold exposure) (Gustafson and Kalkhoff, 1982; Jung et al., 1982), and this research has reported that associations may or not exist between both variables (Breuer et al., 1993). Interestingly, at post-SET, there was a significant improvement in frequency domain variables manifested by an increase in HF and LF with significant decreases in LF/HF ratio, suggesting a possible improvement in sympathovagal balance. The decreases in LF/HF ratio, considered as a sympathetic marker, were associated with noradrenaline, fasting blood glucose, and HOMA-IR decreases without any improvement in waist-to-hip values. Our results bring new insight toward the potential factors involved in ANS modulation/improvement, which have already been studied and confirmed among certain cardiovascular diseases individuals after they completed exercise training.

To elucidate whether obese adults present any inadequate adaptation to the ANS response during exercise, we studied in the current work HRV patterns at VT1 of a graded maximal test. Our result reported a significant higher LF/HF ratio and a lower LF and HF values for obese group than in the normal-weight group. According to previous results, many conflicting responses of frequency domain markers have been reported, and these divergent findings were attributed to varying methodologies (especially HRV analysis techniques) (Scott et al., 2017). For obese individuals, no specific data exist yet regarding the HRV response to exercise. However, these frequency domain measures will help at least to elucidate in what manner these measures that they supposedly reflect**—**namely, cardiac parasympathetic activity (HF) and sympathetic activity or sympathovagal balance (LF and LF/HF ratio)**—**act during exercise exertion. At VT1, the increased values of LF/HF ratio associate significantly with noradrenaline level. The latter appears to reflect higher levels of sympathetic nervous system (SNS) activity, as reported previously by Troisi et al. (1991) and Jones et al. (1997). Therefore, it can be suggested that at submaximal exercise (e.g., VT1 level), increased catecholamine concentration, rather that anthropometric factors, may be a potential marker associated with ANS impairment among obese individuals.

The exercise training significantly decreased adrenaline and noradrenaline responses at the VT1 level and improved the frequency domain marker. The latter is manifested by an increased in LF and HF values and a decreased in LF/HF ratio. LF/HF ratio decreases associated significantly with noradrenaline decreases (β = 0.38, *p* = 0.001). Such adaptation may reflect an improvement in vagal activity and a decrease in sympathetic activity.

Another interesting result reported in our study was the significant decrease in heart rate at rest and at VT1 exertion at post-SET. The heart rate decrease was significantly associated with LF/HF ratio decreases (*r*^2^ = 0.68). For Freitas et al. (2014), an increase in heart rate was observed in obese people and was mainly attributed to impaired autonomic modulation of the intrinsic heart rate, and any improvement in ANS modulation, as observed in the present work, will explain HR decreases in response to SET among obese adults.

This study has limitations that should be acknowledged. Even though no existing method is available today to control the influence of venipuncture procedure on several HRV and considering that in our study both groups have followed the same procedure in terms of blood collection timing (rest and during ventilator threshold 1), we can admit that comparison made between our groups and at pre-post intervention was consistent until “NO” individual differences were established (similar fairness level, anxiety level). Also, while power spectral analysis has been used to quantify cardiac autonomic regulation, accumulating evidence reported that this assumption could be inappropriately interpreted as a major shift toward sympathetic dominance (Houle and Billman, 1999; Billman, 2013). Moreover, it should be noted that power spectral indicators (and thereby LF/HF) were affected by respiratory parameters and mechanical events independent of changes in cardiac autonomic nerve activity. Therefore, addressing other indices of HRV will be helpful in determining the incidence of obesity as well the effectiveness of training program on such parameter. Another limitation of the study is that the sample size is low to derive any meaningful conclusion especially since LF, HF, LF/HF ratio are highly variable. However, a medium effect size has been reported for most of the study outcomes that may supporting at least partially our results. Of course, larger studies are needed to confirm these results. Finally, in the present work, no sample size calculation has been provided to detect difference between the groups. Consequently, the association provided by the present work can be used as a pilot result for which further study is required.

The current study provides several implications for practice. First, while SET seen to be appealing, time efficient and safe for obese adults, the use of SET appears to be efficacious in improving several health indicators, specifically HRV, in obese adults. These improvements observed within 6 weeks offer a significant contribution to health research and practice by developing a new intervention model to promote health benefits among obese adults. Of course, an urgent need is to address a full profile of HRV indices on such study since these outcomes are reliable in terms of finding out the presence of a functional disorder, assessing the level of physical fitness and evaluating the effectiveness of any program intervention and prognosis.

In conclusion, our study suggested that obese adults achieved significant improvements in frequency domain marker and other fitness and hormonal parameters after 6 weeks of SET. The improvements in HRV modulation by the exercise training program correlated with noradrenaline and VO2max increases in obese adults. These finding suggest that changes in HRV is a possible predictor for adaptations to exercise training in obese adults.

## Data Availability Statement

The raw data supporting the conclusions of this article will be made available by the authors, without undue reservation.

## Ethics Statement

The studies involving human participants were reviewed and approved by University’s Human Research Ethics Committee (UHRC) mandated by the Faculty of Higher Studies (under the number: 1415-071). The patients/participants provided their written informed consent to participate in this study.

## Author Contributions

GJ drafted the manuscript, contributed to the conception and design of the study, and to the data collection. GJ and HI performed data analysis and interpretation. Both authors revised, read, and approved the submitted version.

## Conflict of Interest

The authors declare that the research was conducted in the absence of any commercial or financial relationships that could be construed as a potential conflict of interest.
